# Inhibition of histone methyltransferase EZH2 ameliorates early acute renal allograft rejection in rats

**DOI:** 10.1186/s12865-016-0179-3

**Published:** 2016-10-26

**Authors:** Long Li, Yi Zhang, Ming Xu, Ruiming Rong, Jina Wang, Tongyu Zhu

**Affiliations:** 1 Department of Urology, Zhongshan Hospital, Fudan University, Shanghai, 200032 China; 2 Shanghai Key Laboratory of Organ Transplantation, Shanghai, 200032 China; 3 Biomedical Research Center, Institute for Clinical Sciences, Zhongshan Hospital, Fudan University, Shanghai, 200032 China

**Keywords:** Renal transplantation, Acute rejection, Epigenetic regulation, EZH2, DZNep

## Abstract

**Background:**

Although histone methyltransferases EZH2 has been proved to have significant regulatory effect on the immune rejection after hematopoietic stem cell transplantation, its role in solid-organ transplantation remains uncovered. In this study, we investigate whether histone methylation regulation can impact renal allograft rejection in rat models.

**Results:**

Allogeneic rat renal transplantation model (Wistar to Lewis) was established, and the recipients were administrated with EZH2 inhibitor DZNep after transplantation. Renal allografts and peripheral blood were collected on day 5 after transplantation for histological examination and mechanism investigation. We found that inhibition of EZH2 by DZNep after transplantation significantly ameliorated acute rejection (AR), with decreased histological injury and reduced inflammatory infiltration in renal allografts. Attenuation of AR was due to the prohibited activation of alloreactive T cells, the subsequent impaired production of inflammatory cytokines, and also the elevated apoptosis of alloreactive T cells in both renal allografts and periphery. However, inhibition of EZH2 did not increase the regulatory T cells during the AR.

**Conclusions:**

Disruption of EZH2 by DZNep suppressed the immune responses of alloreactive T cells and ameliorated AR of renal allografts. This suggests a therapeutic potential of targeting histone methyltransferases EZH2 in treating allograft rejection after solid organ transplantation.

## Background

In solid organ transplantation, the allogeneic T cells are crucial player during the pathogenesis of immune rejection, since it’s not only the direct mediator of acute cellular rejection, but also synergizes the subsequent humoral rejection [1]. Therefore, comprehensive understanding of the allogeneic T cell regulation is still necessary for the better control of transplantation rejection. In recent years, more and more evidences demonstrated the regulatory effect of epigenetic modifications in T cell immune response [2–7]; however, whether these regulation mechanisms are also involved in the immune rejection is still inconclusive. We and other investigators previously reported the potential of histone methylation in regulating the expression of genes associated with survival, proliferation and differentiation of alloreactive T cells [6, 8], implying the novel epigenetic approaches capable of targeting a specific set of genes in alloreactive T cells may also be useful for controlling T-cell-mediated allograft rejection.

Enhancer of Zeste homology 2 (EZH2), which methylates histone H3 on lysine 27 (H3K27me3), is an essential epigenetic regulator of cell determination and function through histone methylation. Evidence suggests that overexpression of EZH2 is strongly associated with cancer progression and poor outcome in disparate cancers, including hematologic and epithelial malignancies [9]. EZH2 not only facilitate the proliferation, survival and transformation of cancer cells [10, 11], but also impact the tumor immunity [12, 13]. Studies from pharmaceutical company including GlaxoSmithKline and Novartis showed that, EZH2 has already been evaluated as therapeutic cancer target in drug discovery proceeding [14, 15]. Besides cancer cells, stem cells are also under the regulation of EZH2. Disruption of EZH2 results in the shift of mesenchymal stem cell lineage commitment [16], and EZH2 is also associated with the engrafting potential of hematopoietic stem and progenitor cells [17]. Several articles published in 2015 reported the role of EZH2 in T lymphocytes, and found that constraint or deletion of EZH2 dampened T cell differentiation and immune functions both in vitro and in vivo [12, 18]. Ezh2-deficient T effector cells neither provided a protective response to T. *gondii* infection nor mediated autoimmune colitis, and Ezh2-ablation in Treg failed to constrain autoimmune colitis or experimental autoimmune encephalomyelitis [18, 19]. Knockdown of human EZH2 in T cells elicited poor anti-tumor immunity, whereas EZH2^+^CD8^+^ T cells were associated with improved survival in patients [12].

3-deazaneplanocin A (DZNep) is an inhibitor of EZH2. DZNep is recently considered as a potential epigenetic drug, and exerts potent anti-proliferative and pro-apoptotic effects on broad-spectrum carcinomas via disruption of the EZH2 pathway [20]. We first reported DZNep arrested alloreactive T cell-mediated graft-versus-host disease after hematopoetic stem cell transplantation [6, 21]. This effect is associated with the ability of DZNep to selectively deplete EZH2 specific to trimethylation of histone H3K27me3, and activate proapoptotic gene Bim repressed by EZH2 in antigenic-activated T cells [6], and then hamper the pro-inflammatory function of alloreactive T cells without influencing the hematopoietic chimerism and hematopoietic reconstitution [21], revealing the possibilities of controlling immune rejection through the modulation of EZH2, while keeping the functions of allografts.

As we previously reported, renal transplantation is a classic model of solid organ transplantation, and acute renal allograft rejection is actually a kind of inflammatory response with significant accumulation and activation of monocytes, especially T lymphocytes in the graft [22]. Therefore in this study, the renal transplantation model were elected to investigate the effect of EZH2 and DZNep in allogeneic T-cell-mediated acute rejection (AR) in solid organ transplantation. We observed elevated EZH2 in T cells after allogeneic renal transplantation, and disruption of EZH2 by DZNep attenuated acute renal allograft rejection, with ameliorated tissue injury and inflammatory infiltration in the renal allograft. The cellular mechanisms are related to the prohibited activation and proliferation of alloreactive T cells, the subsequent impaired inflammatory cytokine production, and also the elevated apoptosis of alloreactive T cells in both renal allografts and periphery. However, no promoted Tregs was observed with the inhibition of AR by EZH2 disruption. It is the first time we proved the relationship of EZH2 and the immune rejection after allogeneic renal transplantation, and suggested the potential of pharmacologic inhibition of EZH2 by DZNep as a novel strategy for treating rejection after solid organ transplantation.

## Methods

### Experimental animals

Lewis rats were purchased from Beijing Vital River Laboratory Animal Technology Company (Beijing, China). Wistar rats were purchased from SLAC Laboratory Animal Co., Ltd (Shanghai, China). Animals were housed in specific pathogen-free facility at the Zhongshan Hospital, Fudan University. All experimental protocols were approved by Committee of Zhongshan Hospital, Fudan University on Use and Care of Animals.

### Flow cytometric analysis

Mononuclear cells (MNCs) of peripheral blood were obtained by Ficoll density gradient centrifugation and analyzed by flow cytometry. Monoclonal antibodies with the following specificities were obtained from BD Biosciences or eBioscience, or BioLegend (San Diego, CA, USA): CD4 (OX-38), CD8 (OX-8), CD25 (OX-39), and Foxp3 (FJK-16 s). Foxp3/Transcription Factor Staining Buffer Set was used for intracellular staining Multiple-color flow cytometry analysis was performed using FACS Aria (BD Biosciences).

### Rat model of renal transplantation

Rats at 8–10 weeks, 180–220 g body weight were used for transplantation. The transplantation was processed as described previously [22]. Briefly, the donor kidney removed from a Wistar or Lewis rat was transplanted orthotopically into a unilaterally nephrectomized Lewis recipient with end-to-end anastomosis of renal artery, vein and ureter. Warm ischemia time did not exceed 40 min. No immunosuppressant were used in recipients. The construction of rat renal transplantation model was considered a success if the transplanted animal survived for at least 4 days. DZNep was bought from the Cayman Chemical Company (Ann Arbor, MI, USA). Recipient rats were divided into three groups (*n* = 5 for each group): (1) Isogenic group, rats transplanted with isografts (Lewis to Lewis); (2) Allogeneic group, rats transplanted with allografts (Wistar to Lewis) and with phosphate buffer (PBS) treatment; (3) DZNep group, rats transplanted with allografts (Wistar to Lewis) and with DZNep treatment (1 mg/kg/d DZNep once per day after transplantation from day 0 to day 4, subcutaneous injection).

### Histologic analysis of renal allograft rejection

Paraffin sections of formalin-fixed renal grafts were stained with hematoxylin and eosin (H&E), and were reviewed for inflammatory infiltrating and tubule injury. Tissues were graded using the Banff 97 grading system by two pathologists who were blinded to the diagnosis. The following Banff components were evaluated: glomerulitis (g), tubulitis (t), interstitial inflammation (i), arteriola hyalinosis (ah), and intimal arteritis (v). Glomerulitis (g) was scored as g0-g3: 0 %, <25 %, 25–75 %, and >75 % of glomeruli, respectively; Tubulitis (t) was scored as t0-t3: 0, foci with 1–4, foci with 5–10, foci with >10 cells/tubular cross section, respectively; Interstitial inflammation (i) was scored as i0-i3: <10 %, 10–25 %, 26–50 %, >50 % of parenchyma inflamed, respectively; Arteriola hyalinosis (ah) was scored as ah0-ah3: no, mild-to-moderate, moderate-to-severe, severe PAS-positive hyaline thickening, respectively; Intimal arteritis (v) was scored as v0-v3: no, mild-to-moderate, severe intimal arteritis, arterial fibrinoid change and/or transmural arteritis with medial smooth muscle necrosis with lymphocytic inflammation, respectively.

### Immunohistochemistry (IHC)

Immunohistochemical staining of CD4, CD8, and CD68 (Abcam, Cambridge, UK) was performed on paraffin-embedded or frozen sections using a DAKO ChemMate EnVision Detection Kit (DAKO, Carpinteria, CA, USA) as described previously [22]. Semi-quantification for IHC staining slides were performed under high power filed (HPF, 400×).

### Detection of apoptosis

Terminal deoxynucleotidyl transferase-mediated dUTP-biotin nick end labeling (TUNEL) staining kit (Millipore, MA, USA) was used to detect apoptotic cells in renal grafts, and the manufacturer’s instructions were followed. Semi-quantification for apoptotic cells were performed under high power filed (HPF, 400×).

### Western blot

CD3^+^CD25^+^ T cells were sorted out from MNCs by flow cytometry. The purity of sorted cells in this study was consistently more than 99 %. Then, cells were directly lysed with RIPA containing protease and phosphatase inhibitors (Roche Applied Science, Indianapolis, IN, USA) and proteins were separated by 10 % SDS-PAGE after denaturation. Immunoblot analysis was performed by initial transfer of proteins onto polyvinylidenefluoride membranes using Mini Trans-Blot (Bio-Rad Laboratories, Richmond, VA, USA) and followed by a blocking step with 5 % nonfat dried milk plus 0.1 % Tween 20 for 2 h at room temperature and exposed to primary antibodies diluted 1000-fold that recognized EZH2 and actin overnight at 4 °C and subsequently washed. The blots were then incubated with a secondary antibody conjugated with Horse Radish Peroxidase diluted 5000-fold for 1 h at room temperature. Signals were detected by FluorChem E system (Alpha Innotech Corp, Santa Clara, CA, USA).

### Cytokine analysis

The levels of IFN-γ, TNF-α, IL-2, IL-17, IL-4 and IL-10 in plasma were assessed with enzyme-linked immunosorbent assay (ELISA) kits according to the manufacturer’s instructions (R&D Systems, Minneapolis, MN, USA).

### Statistical analysis

Data presented as means ± SEM, and Kruskal-Wallis test was used for statistics among the three groups. Differences were considered statistically significant if the *p* value was less than 0.05.

## Results

### Inhibition of EZH2 by DZNep ameliorated acute renal allograft rejection

**Fig. 1:**
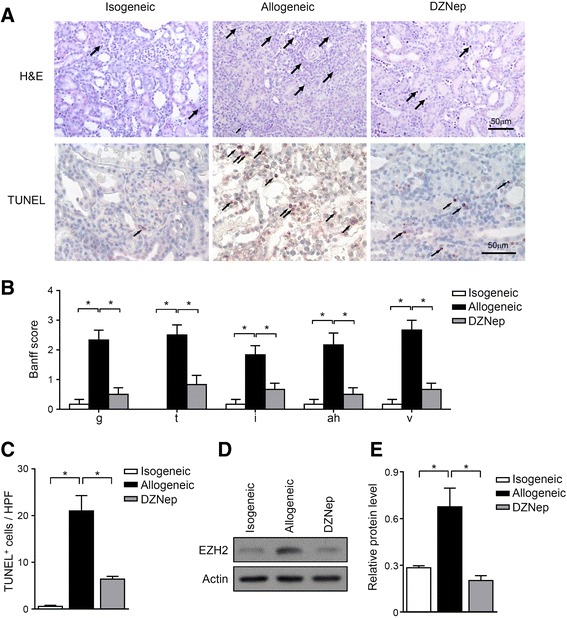
Attenuated acute renal allograft rejection by EZH2 inhibition. Rat renal transplantation was performed as described in methods. Groups are classified in Methods. Renal grafts and recipient periphery blood were collected on day 5 after transplantation for analysis. (**a**) H&E and TUNEL staining were performed for renal grafts. (**b**) Histochemical quantitative data of Banff components including glomerulitis (g), tubulitis (t), interstitial inflammation (i), arteriola hyalinosis (ah), and intimal arteritis (v) was evaluated. (**c**) Semi-quantification apoptotic cells in renal graft parenchyma were examined. (**d**, **e**) EZH2 protein levels in peripheral CD3^+^CD25^+^ T cells were examined by western blot (**d**), and quantification was done by densitometry of western blot bands (normalized to actin) (**e**). *, *p* < 0.05, Kruskal-Wallis test

Rat renal transplantation was performed and the recipients were sacrificed on day 5 after transplantation. Since in this study, recipient rats were processed by unilaterally nephrectomy and orthotopical transplantation, the survival status and urine data would not represent the functions of allografts. Histological examination showed that the acute graft rejection occurred in allogeneic group, as evidenced by acute tubular injury, diffuse heavy inflammatory infiltration with severe glomerulopathy, intimal arteritis and arteriola hyalinosis. However, when treated with DZNep, the allograft tissue damage and inflammatory infiltration were remarkably attenuated (Fig. 1a and b). The TUNEL staining also showed that, allografts without DZNep treatment had more apoptotic cells, which mainly located on tubular and interstitial areas, and the DZNep treatment also reduced the apoptotic cells in allografts (Fig. 1a and c), suggesting that administration of DZNep can protect allografts with reduced tissue inflammation and damages. Further, in order to confirm the role of EZH2 inhibition by DZNep in renal transplantation, the protein levels of EZH2 were determined by western blot. As we expected, the declined EZH2 in the alloreactive T cells were observed in DZNep-treated recipients (Fig. 1d and e). All those data demonstrated that in rat renal transplantation, disruption of EZH2 by DZNep ameliorated acute renal allograft rejection.

### DZNep treatment prohibited T cell infiltrating in allograft

**Fig. 2:**
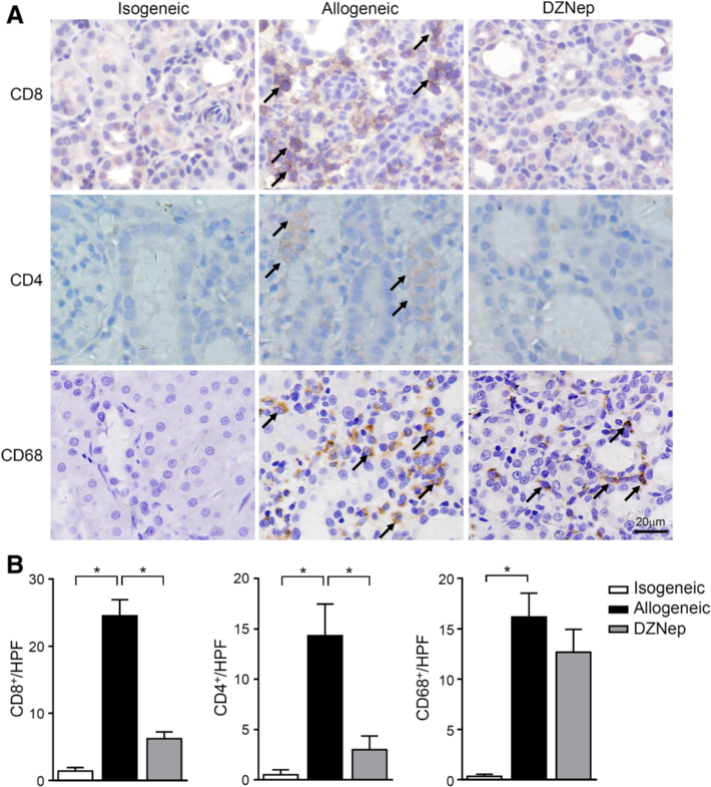
Diminished alloreactive T cell infiltration with DZNep treatment. IHC staining of CD4, CD8 and CD68 on renal grafts (**a**), and the semi-quantification of CD4^+^ and CD8^+^ lymphocytes and CD68^+^ macrophages (**b**). *, *p* < 0.05, Kruskal-Wallis test

We next investigated the mechanisms by which DZNep protects the renal grafts. Since alloreactive T cells are the direct mediator of acute rejection, their infiltrating status in renal grafts was then detected by IHC. We found that, renal grafts in allogeneic transplantation had elevated CD4 and CD8 staining as compared with those in isogeneic transplantation, while DZNep treatment in allogeneic transplantation prohibited the elevation (Fig. 2a). Semi-quantification analysis further confirmed the significance of T cell infiltrating differences when DZNep was applied (Fig. 2b). However, although the infiltration of CD68^+^ macrophages in allograft was increased, it seems that DZNep treatment did not significantly suppress the macrophage infiltration (Fig. 2a and b). These data suggested that DZNep can inhibit the accumulation of alloreactive T cells in allografts, and also implying the possibility of impacted T cell response in DZNep administration.

### DZNep blemished the activation and survival of alloreactive T cells

**Fig. 3:**
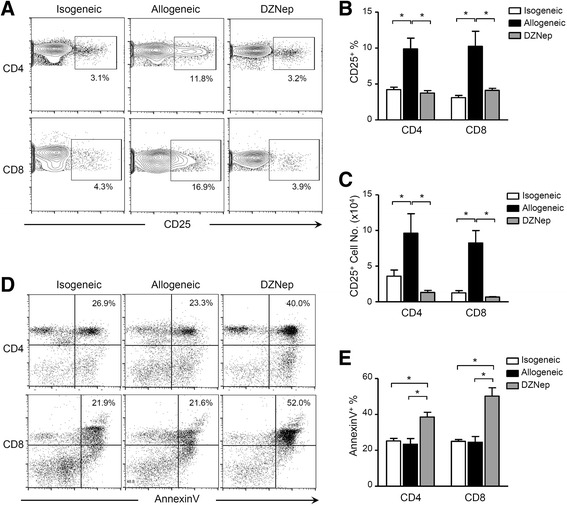
DZNep inhibits the survival of alloreactive T cells. Peripheral blood was harvested from recipient rats on day 5 after transplantation for flow cytometry analysis. Percentages of peripheral CD4^+^CD25^+^ and CD8^+^CD25^+^ cells were determined (**a** and **b**). The absolute numbers of peripheral CD4^+^CD25^+^ and CD8^+^CD25^+^ cells were also calculated (**c**). Percentages of Annexin V^+^ CD4^+^ and Annexin V^+^ CD8^+^ were presented (**d** and **e**). *, *p* < 0.05, Kruskal-Wallis test

The alloreactive T cell response includes the activation in response to allo-antigens and the survival after activation. Therefore, the activation and apoptosis status of recipient T cells were then tested by flow cytometry. On day 5 after transplantation, recipients with DZNep treatment showed lower frequency of CD25 expression on both CD4^+^ and CD8^+^ T cells, as compared with those in allogeneic group (Fig. 3a and b), and also had reduced absolute numbers of activated T cells in periphery (Fig. 3c), indicating that DZNep are able to inhibit T cell activation in recipients. On the other hand, increased AnnexinV^+^ proportions in both CD4^+^ and CD8^+^ T cells were observed in DZNep-treated recipients, as compared with those in allogeneic group (Fig. 3d and e), suggesting a pro-apoptotic role of DZNep in alloreactive T cells. These data suggest that, EZH2 are promotive for alloreactive T cell response and DZNep can blemish the activation and survival of alloreactive T cells after renal transplantation.

### DZNep ameliorated local and systemic inflammatory environment

**Fig. 4:**
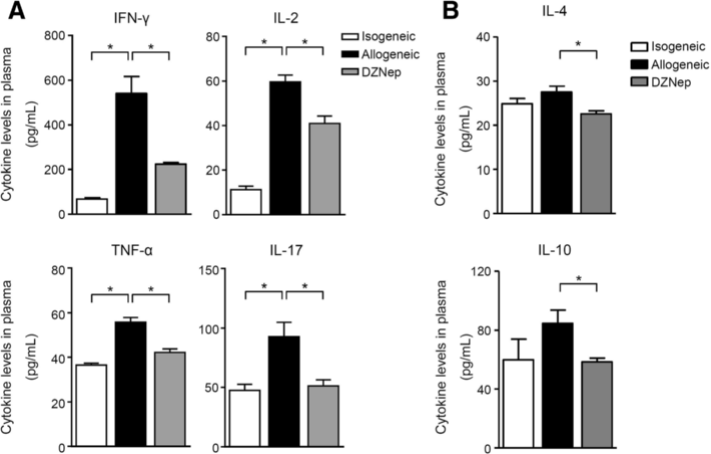
DZNep inhibits inflammatory cytokines in periphery. **a**, **b** ELISA assays were performed to test the plasma cytokine levels on day 5 after transplantation. IFN-γ, TNF-α, IL-17, and IL-2 levels were presented (**a**), IL-4 and IL-10 levels were shown (**b**). *, *p* < 0.05, Kruskal-Wallis test

Alloreactive effector T cells mediate allograft injury through direct contact with tubular epithelial cells (cell-mediated cytotoxicity) and locally released cytokines. We have demonstrated that DZNep ameliorated local inflammation in renal grafts with less CD4^+^ T and CD8^+^ T cells infiltration in the interstitial area of renal allografts (Fig. 2a and b). We also tested the inflammatory cytokine expression in peripheral blood by ELISA. Typical pro-inflammatory cytokines, such as TNF-α, IFN-γ, IL-17, and IL-2, which can drive cellular response, impair the allograft function and intensify T-cell mediated rejection after allogeneic organ transplantation [23, 24], were found to be significantly reduced in DZNep-treated recipients, in contrast to those in allogeneic group after renal transplantation (Fig. 4a). Surprisely, we also found that the levels of IL-4 and IL-10 were also declined by DZNep administration (Fig. 4b), which implied that inhibition of EZH2 by DZNep did not function through elevating Th2 response to suppress Th1 response. The controversial roles of IL-4 and IL-10 in renal rejection are discussed in the section of Discussion. Taken together, these data suggest that DZNep can control the local and systemic inflammatory environment, which is promotive for renal graft survival and function.

### DZNep protected renal allografts through a Treg-irrelevant pathway

**Fig. 5:**
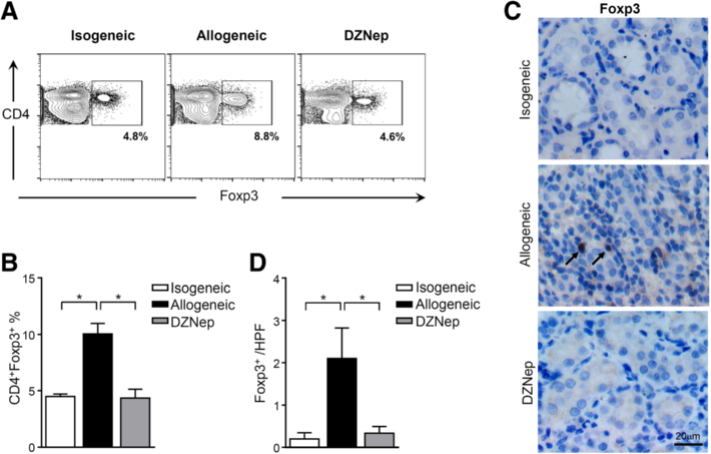
DZNep does not increase the differentiation of Tregs. The percentages of peripheral CD4^+^Foxp3^+^ were examined by flow cytometry (**a** and **b**). IHC staining of Foxp3 were performed on renal grafts (**c**), IHC semi-quantification of Foxp3^+^ cells were analyzed (**d**). *, *p* < 0.05, Kruskal-Wallis test

Regulatory T cells (Tregs) that express the transcription factor Foxp3 are a highly suppressive population, and are considered to be critical for the maintenance of tolerance to self-components [25]. Some reports suggested that the induction of immunologic tolerance of organ transplantation was characterized by reduction of alloreactive effector T cells and increased numbers of Tregs [26, 27]. However, in this study, we did not observe increased Tregs with DZNep treatment after transplantation. In contrast, peripheral CD4^+^Foxp3^+^ Tregs in DZNep-treated recipients was declined when compared to recipients with AR (Fig. 5a and b), and in renal grafts, diminished Foxp3^+^ T cell infiltration was also observed when DZNep was applied to recipients (Fig. 5c and d). These findings imply that the suppression of acute renal rejection by DZNep is not mediated through the immunosuppression functions of Treg, in contrast, inhibition of EZH2 by DZNep blemished recipient T cell response which may also include the Treg differentiation after transplantation.

## Discussion

Lifelong immunosuppression is associated with severe side effects, such as diabetes, cardiovascular diseases, cancer, infection, and allograft toxicity, thereby limiting long-term allograft survival [28]. Therefore, better understanding of the allogeneic immune response and identification of new therapeutic targets are still needed. Here we identify that histone methyltransferase EZH2 is critical for alloreactive T cell response after allogeneic renal transplantation, and inhibition of EZH2 by DZNep can protect the allograft from inflammatory infiltration and promote graft survival.

Although EZH2 and its epigenetic modification function have already been considered to be important for T-cell responses, it’s still the first time to directly demonstrate that EZH2 is involved in the pathogenesis of solid organ transplantation and promotes acute allogeneic rejection. In rat transplantation model, disruption of EZH2 by DZNep suppressed alloreactive T cell activation, limited their survival, and controlled the local and systemic inflammatory environments.

The role of inflammatory cytokines and particularly the role of Th2 cytokines IL-4 and IL-10 in the course of allograft acute rejection are subjected of intensive research for decades, but now are still controversial. Increased levels of IL-4 and IL-10 were observed in the immune rejection courses of several organs and tissues, including skin and kidney [29, 30]. In animals, IL-4 mediated tissue allograft eosinophil infiltration is associated with interstitial fibrosis [29], and induction of a Th2 allogeneic response resulted in *de novo* development of chronic rejection [31]. In patients, higher levels of IL-4 prior to and shortly after transplantation may have protective effects on graft survival. However, a prolonged, increased production of IL-4 after transplantation also contribute to acute allograft rejection episodes [32]. Although IL-10 has been shown to have the suppressive function on antigen-specific effector cell responses [33], it was also reported as a stimulator of the immune system, inducing the differentiation and proliferation of B cells, thus leading the immune response toward the humoral pathway and enhancing antibody responses against the graft [34]. Here DZNep treatment can significantly reduce the levels of both IL-4 and IL-10, so we can conjecture that DZNep may be able to further suppress the induction of chronic renal rejection, delay the course of interstitial fibrosis and benefit long-term allograft survival.

It is interesting that DZNep treatment resulted in less CD4^+^Foxp3^+^ Tregs in peripheral blood and renal allografts. This coincides with our previous observations that DZNep treatment decreased both frequency and number of peripheral Tregs during GVHD [6]. This phenomenon can be explained by two aspects. First, in the present study, DZNep treatment not only prohibits the infiltration of T cell in allograft, but also suppresses the activation and survival of alloreactive T cells in the periphery, implying that DZNep induces apoptosis of activated T cells in the lymph node and no activated lymphocytes in the periphery are able to home in the allograft. Therefore, the lack of inflammatory cells in blood and allograft might explain the lack of Tregs at that site and low levels of both Th1 and Th2 cytokines in the plasma. These results are consistent with the study showing that deficiency of EZH2 dampened Th differentiation and in Treg was associated with a defect in Treg stability, and EZH2 may promote the maintenance of Foxp3 expression during Treg cell responses [18, 19]. Another, although evidence accumulated in past decade supports the critical role of Tregs in the suppression of alloimmune responses, there’s still conflicting results emerged from many studies and may have generated more confusion than clarification. Several studies showed poor correlation between Tregs infiltration and allogeneic graft status, based on Foxp3 analysis from graft biopsy cores, some studies confirmed higher Foxp3 expression in the grafts exhibiting cellular acute rejection [35, 36]. It is also reported that Tregs are first activated in the allograft, and subsequently migrate to the draining lymph nodes and peripheral blood, where they suppress the alloantigen-specific immune response [37]. These findings are helping explain higher Foxp3-expressing Treg-levels in peripheral blood and allografts in recipient rats with acute rejection. Taken together, these observations suggest that the protective effect of DZNep on renal allografts was realized through a pathway irrelevant of increased Tregs in peripheral blood and renal allografts.

In this study, we transplanted renal grafts orthotopically into unilaterally nephrectomized recipient rats, that’s why we did not show the survival curves of graft/recipient rats. Although renal allograft function in terms of serum creatinine and urine nitrogen levels are also invalid for evaluation, the pathophysiological process of AR and histological change of renal grafts are not influenced. There are many merits to use unilaterally nephrectomized recipients model. First, this model is also well qualified for investigating the allogeneic immune response, including the pathology of allografts and allogeneic T cells responses. Second, unilaterally nephrectomized recipient possess a better post-transplant condition compared to bilaterally nephrectomized recipients, especially recipients with delayed graft function, so as to enhance the steadiness of renal transplant model.

Based on our and other research results, DZNep is different from traditional immunosuppressive agents such as cyclosporine A and tacrolimus. The first reason is the reversibility of DZNep’s effect [38]. Secondly, DZNep possesses broad potent antiviral activity, including against rotavirus, vesicular stomatitis virus and vaccinia virus [39, 40]. In particular, this antiviral spectrum of DZNep extends to human cytomegalovirus, which is capable of causing serious infection in transplanted recipients with reduced immune functions [40]. Thirdly, although pharmacological EZH2 inhibition by DZNep shows broad anti-tumor effect in several cancers, including but not limited to prostate cancer, breast cancer, acute myeloid leukemia, and particular renal cell carcinoma [41–44]. All those data imply that DZNep may not be limited to an immunosuppressive agent for treating allografts rejection, but also a potential drug for preventing/treating virus infections and malignant tumors after organ transplantation. However, the side effects should be brought to consideration as well. As we introduced previously, EZH2-abalation affects the regulatory T cell generation, increased the risk of infection, and also dampen the immune response against cancerous cells [12, 18, 19]. Therefore, more investigations should be processed to carefully evaluate the effects of DZNep before clinical application.

## Conclusions

To summarize, we have demonstrated that inhibition of histone methyltransferase EZH2 by DZNep can protect renal allograft from acute rejection. It is the first time we demonstrated the therapeutic function of DZNep in solid organ transplantation, and there are still more investigations required for comprehensive understanding of mechanisms. For instance, the impact of DZNep on other immune cells, and how to optimize the DZNep treatment, are all needed to develop DZNep for clinical use.

## Abbreviations

AR: Acute rejection
DZNep: 3-deazaneplanocin A
ELISA: Enzyme-linked immunosorbent assay
EZH2: Enhancer of Zeste homology 2
H&E: Hematoxylin and eosin
HPF: High power filed
IFN: Interferon
IHC: Immunohistochemistry
IL: Interleukin
PBS: Phosphate buffer
TNF: Tumor necrosis factor
Treg: Regulatory T cells
